# Efficient and real-time polyp segmentation: An empirical evaluation of lightweight Mamba integration strategies

**DOI:** 10.1371/journal.pone.0353562

**Published:** 2026-10-09

**Authors:** Si Chen, Mohd Shafry Mohd Rahim, Hiew Moi Sim, Yee Yong Pang, Pei Kian Teo, Ei Lin Ong, Wei Jer Lim

**Affiliations:** 1 Faculty of Computing, Universiti Teknologi Malaysia, Johor Bahru, Malaysia; 2 School of Artificial Intelligence, Guangzhou Institute of Science and Technology, Guangzhou, China; 3 Faculty of Art and Design, Southern University College, Johor Bahru, Malaysia; 4 Faculty of Engineering and Information Technology, Southern University College, Johor Bahru, Malaysia; Sichuan University, CHINA

## Abstract

Accurate and efficient polyp segmentation is essential for computer-aided colorectal cancer screening, yet achieving a favorable balance among segmentation accuracy, computational efficiency, and cross-dataset generalization remains challenging. Recent studies suggest that Mamba-based state-space modeling can enhance long-range dependency modeling with relatively low complexity, but how lightweight Mamba integration strategies affect dense prediction frameworks for polyp segmentation remains insufficiently understood. In this study, we present LiteMamba-Seg, a lightweight polyp segmentation framework built on a U-Net-like encoder–decoder architecture with a ResNet34 backbone, and conduct an empirical evaluation of different lightweight Mamba integration strategies. Specifically, we investigate bottleneck-only integration as well as alternative second-stage placements at an encoder-side intermediate stage and a decoder-side reconstruction stage. Experimental results on four public benchmark datasets show that LiteMamba-Seg achieves competitive segmentation performance while maintaining a lightweight design and real-time model-level inference, with 26.81 M parameters, 13.89 GFLOPs, and 167.73 FPS. Further ablation, controlled placement, multi-seed, robustness, boundary, calibration, and false-positive analyses indicate that the effect of Mamba integration depends on its placement within the network. In particular, decoder-side reconstruction-stage integration yields more favorable quantitative and qualitative results than encoder-side intermediate placement across the evaluated benchmarks. These findings suggest that, for lightweight polyp segmentation frameworks, Mamba integration should be considered jointly with placement strategy rather than simply increasing the number of sequence modeling blocks.

## Introduction

Colorectal cancer remains one of the leading causes of cancer-related mortality worldwide. Since most colorectal cancers arise from precancerous adenomatous polyps, accurate detection and delineation of polyps during colonoscopy are essential for early intervention and improved clinical outcomes. In recent years, computer-aided detection systems have shown substantial clinical potential in assisting endoscopists during routine examinations and improving lesion detection rates [1,2] Among related tasks, polyp segmentation is particularly important because it provides pixel-level localization of lesion extent, which is valuable for lesion boundary assessment, size estimation, lesion monitoring, and subsequent clinical decision-making [3].

Despite substantial progress, automatic polyp segmentation remains challenging in real-world colonoscopy scenarios. Polyps vary considerably in size, shape, texture, and appearance, and difficult cases often involve small lesions, low contrast against surrounding mucosa, irregular morphology, or blurry boundaries [4]. In addition, specular highlights, motion blur, intestinal contents, and illumination variation can further obscure lesion regions and degrade prediction quality [5]. These challenges become more pronounced when models are evaluated on unseen datasets collected under different imaging conditions, where domain shift often leads to a clear drop in segmentation performance [6]. Therefore, an effective polyp segmentation framework should not only achieve high accuracy on in-domain distributions, but also maintain reliable performance across different domains.

Existing polyp segmentation frameworks have achieved encouraging progress, yet balancing segmentation accuracy, computational efficiency, and cross-domain robustness remains a persistent challenge. In practical deployment, a segmentation framework should be not only accurate, but also lightweight, efficient, and reliable under unseen imaging conditions. In the broader deep learning literature, lightweight architecture design has been widely explored to improve deployment efficiency, as exemplified by models such as MobileNetV3 [7] and GhostNet [8]. More recently, selective state space models (SSMs), especially Mamba and its medical variants, have emerged as an efficient alternative for long-range dependency modeling, and recent review studies have highlighted their growing role in medical image analysis [9]. Representative frameworks such as U-Mamba [10] further show how state-space modeling can be incorporated into biomedical dense prediction pipelines. Built on these developments, subsequent lightweight Mamba-based designs have continued to explore parameter-efficient segmentation mechanisms [11–13]. However, how such state-space capability should be integrated into a lightweight hierarchical pipeline remains insufficiently studied for polyp segmentation. In particular, the effect of lightweight Mamba block placement, especially whether such modules are more effective at encoder stages or decoder reconstruction stages, has not been sufficiently investigated.

Motivated by these considerations, we develop LiteMamba-Seg, a lightweight segmentation framework for efficient polyp segmentation with an emphasis on cross-dataset evaluation. Built on a U-Net-like encoder–decoder architecture with a ResNet34 backbone, LiteMamba-Seg introduces Mamba-based contextual modeling in a selective rather than densely stacked manner, aiming to enhance semantic representation while keeping additional complexity limited. More importantly, instead of treating Mamba as a plug-in component that can be inserted arbitrarily, we investigate how different lightweight Mamba integration strategies affect segmentation performance, computational efficiency, and cross-dataset generalization. This framework serves not only as an efficient segmentation model, but also as a practical basis for studying how lightweight contextual modeling should be integrated for dense prediction. The main contributions of this work are summarized as follows:

1. We develop LiteMamba-Seg, a lightweight segmentation framework for efficient polyp segmentation.
2. We conduct an empirical evaluation of lightweight Mamba integration strategies and analyze how different component choices and placement schemes affect segmentation behavior.
3. We validate the proposed framework on four public benchmark datasets and demonstrate competitive performance under both standard in-domain and cross-dataset evaluation settings.
4. We conduct detailed analyses on computational efficiency, component behavior, Mamba placement, multi-seed stability, input perturbation robustness, boundary quality, calibration, and false-positive behavior to examine the practicality and reproducibility of the proposed design.

## Related work

Recent advances in polyp segmentation can be broadly categorized into CNN-based architectures, Transformer-based models, and, more recently, Mamba-enhanced designs. In this section, we briefly review these representative directions and highlight the remaining research gap that motivates the present study.

### CNN-based polyp segmentation

Convolutional neural networks (CNNs) have long been widely used in medical image segmentation because of their strong local representation ability and favorable computational efficiency. Classical encoder–decoder architectures such as U-Net [14] and UNet++ [15] established an important foundation for biomedical dense prediction by combining hierarchical feature extraction with multi-scale skip connections.

In polyp segmentation, CNN-based frameworks such as PraNet [16] and ACSNet [17] further improved lesion delineation through reverse attention mechanisms and multi-scale contextual aggregation. To support practical deployment, recent lightweight architectures such as LightCF-Net [18] have also explored how to preserve segmentation accuracy while reducing computational cost for real-time colonoscopy applications. However, although CNNs are effective at modeling local textures and short-range spatial patterns, capturing broader contextual dependencies may require deeper architectures or additional contextual modeling mechanisms.

### Transformer-based segmentation

To address the limitations of restricted receptive fields, Transformer-based methods have been introduced into medical image segmentation [19]. By using self-attention to model long-range spatial dependencies, these methods provide an effective mechanism for global contextual modeling beyond conventional convolutional networks [20]. Representative hybrid frameworks such as TransUNet [21] and TransFuse [22] showed that Transformer-enhanced feature modeling can complement convolutional representations in dense prediction tasks.

For polyp segmentation, methods such as Polyp-PVT [23] further demonstrated the potential of Transformer-based feature encoding for lesion analysis. More recent hybrid designs have continued to explore richer feature fusion strategies. For example, DECTNet [24] combines a convolution-based encoder with a Swin Transformer encoder for hierarchical representation learning, while DSCANet [25] adopts a dual-encoder framework with spatial cross-attention for polyp segmentation. These studies further highlight the value of combining local feature extraction with stronger contextual modeling.

However, Transformer-based and dual-stream attention architectures can introduce considerable computational and memory overhead, particularly when processing high-resolution images. This motivates continued investigation of more computationally efficient approaches for polyp segmentation.

### Mamba-based segmentation

More recently, selective SSMs, especially Mamba and its visual variants, have emerged as an efficient alternative for long-range sequence modeling [26]. Optimal transport-based undersampling preserves data distributions under class imbalance using Wasserstein distance [27], while lightweight Mamba variants emphasize efficiency and deployability [12,28]. Related efforts have also explored combining state-space modeling with additional spatial modules to better fuse global dependencies and local representations, as exemplified by SA-UMamba [29].

Recent Mamba-enhanced studies on polyp segmentation have explored different architectural strategies. For example, ProMamba [30] investigates prompt-based feature enhancement, BAMPolyp [13] introduces a bi-axial Mamba bottleneck for gastrointestinal polyp segmentation, and PolyMamba [31] incorporates spatial-prior guidance and high-frequency enhancement to better adapt to complex lesion morphology. These studies suggest that Mamba-based state-space modeling can complement convolutional feature extraction for polyp segmentation. However, many existing designs focus primarily on proposing new Mamba-enhanced architectures, while comparatively less attention has been given to how the placement and integration strategy of lightweight Mamba blocks affect segmentation performance within hierarchical 2D encoder–decoder networks. Therefore, the influence of lightweight Mamba integration strategy and placement remains insufficiently understood in polyp segmentation.

## Methodology

This section describes the LiteMamba-Seg framework in detail. We first introduce the overall network architecture, followed by the ResNet34-based hierarchical encoder, the lightweight Mamba-based context modeling block, and the decoder for progressive feature reconstruction. Finally, the efficiency-oriented design is described to explain how LiteMamba-Seg maintains a lightweight computational profile.

### Overall architecture

LiteMamba-Seg is a lightweight polyp segmentation framework built on a U-Net-like encoder–decoder architecture [14]. As shown in Fig 1, the network consists of three main components: a pre-trained ResNet34-based encoder [32] for hierarchical feature extraction, lightweight Mamba-based context modeling blocks [11], and a progressive decoder with deep supervision for spatial reconstruction.

**Fig 1:**
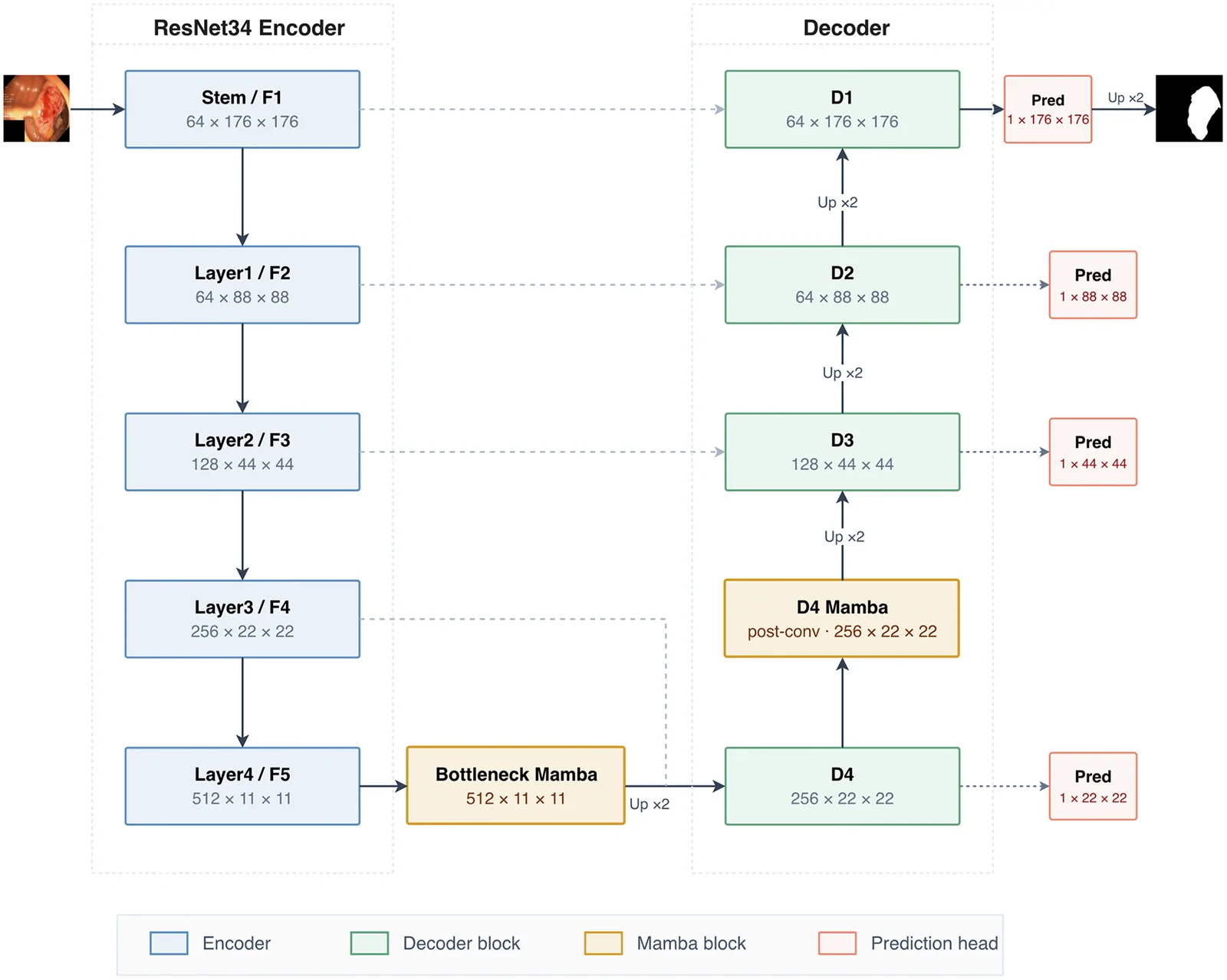
Overall architecture of LiteMamba-Seg. The prediction from $D_{\text{1}}$ serves as the main output, whereas the predictions from $D_{\text{2}}$-$D_{\text{4}}$ are used as auxiliary supervision during training.

Given an input colonoscopy image $I\in R^{H\times W\times \text{3}}$, the encoder progressively extracts hierarchical multi-stage feature representations through an initial convolutional stem and four subsequent residual stages. Let $F_{\text{1}},F_{\text{2}},F_{\text{3}},F_{\text{4}},F_{\text{5}}$ denote the feature maps produced by the stem, Layer1, Layer2, Layer3, and Layer4 of the ResNet34 backbone, respectively. Their channel dimensions are:


$$\begin{array}{c} C_{i}\in \{\text{6}\text{4},\text{6}\text{4},\text{1}\text{2}\text{8},\text{2}\text{5}\text{6},\text{5}\text{1}\text{2}\},\;i\in \{\text{1},\text{2},\text{3},\text{4},\text{5}\} \end{array}$$ (1)

Shallow features ($F_{\text{1}},F_{\text{2}}$) are generally associated with low-level structures such as lesion boundaries and texture patterns, whereas deeper features ($F_{\text{3}},F_{\text{4}},F_{\text{5}}$) provide increasingly compact and abstract representations.

On top of this hierarchical representation, LiteMamba-Seg introduces lightweight Mamba-based global context modeling at two key stages of the network. In the main architecture, one Unified Mamba Block is applied to the deepest encoder feature $F_{\text{5}}$ as the bottleneck transformation, while another is placed after the first decoder reconstruction stage ($D_{\text{4}}$). This dual-stage design introduces contextual modeling at the deepest encoder stage and again during early decoder reconstruction. In addition, for ablation purposes, we also evaluate an alternative encoder-side placement at Layer3 to investigate the stage-dependent behavior of Mamba integration. These locations were selected as representative integration stages under the lightweight design objective of LiteMamba-Seg rather than as an exhaustive search over all possible network positions. $F_{\text{5}}$ represents the deepest low-resolution encoder feature, Layer3 provides an intermediate encoder-side alternative, and $D_{\text{4}}$ represents the first decoder reconstruction stage. Higher-resolution stages were not exhaustively evaluated because the purpose of this study was to examine representative lightweight integration configurations rather than dense Mamba insertion throughout the network.

Following feature extraction and context modeling, the decoder progressively restores spatial resolution through multi-stage transposed-convolution upsampling. At each decoding stage, the upsampled features are concatenated with the corresponding encoder features through skip connections to recover structural details lost during downsampling. Transposed convolution was adopted for learnable upsampling while maintaining alignment with the corresponding skip features. To improve gradient propagation and multi-scale learning, LiteMamba-Seg further adopts a deep supervision mechanism during training [33]. The overall end-to-end mapping can be written as:


$$\begin{array}{c} \{\hat{M_{\text{1}}},\hat{M_{\text{2}}},\hat{M_{\text{3}}},\hat{M_{\text{4}}}\}=F_{\text{LiteMamba}-\text{Seg}}(I) \end{array}$$ (2)

where $F_{\text{LiteMamba}-\text{Seg}}(\cdot )$ denotes the overall network mapping, and $\hat{M_{g}}\in R^{H\times W\times \text{1}}$ (g ∈{1,2,3,4}) denotes the set of predicted segmentation masks from different decoder stages. During inference, only the highest-resolution prediction $\hat{M_{\text{1}}}$ is retained as the final segmentation output. Through this encoder–decoder design, LiteMamba-Seg combines hierarchical convolutional representation learning with selective Mamba-based contextual modeling in a compact architecture.

### ResNet34 encoder

Within the proposed architecture, the encoder serves as the foundation for hierarchical feature extraction and provides the local structural representations upon which subsequent Mamba-based global modeling is built. For this purpose, LiteMamba-Seg adopts a pre-trained ResNet34 [32] as the backbone, since it offers a favorable balance between representational capacity and computational efficiency, providing a favorable balance between representation capacity and computational efficiency.

Given an input colonoscopy image $I\in R^{H\times W\times \text{3}}$, the encoder progressively transforms it into hierarchical feature maps through an initial convolutional stem followed by four successive residual stages:


$$\begin{array}{c} \left(F_{\text{1}},F_{\text{2}},F_{\text{3}},F_{\text{4}},F_{\text{5}}\right)=E(I) \end{array}$$ (3)

Here, $F_{\text{1}}$ denotes the initial feature map generated by the convolutional stem, while $F_{\text{2}},F_{\text{3}},F_{\text{4}}$ and $F_{\text{5}}$correspond to the output feature representations of Layer1, Layer2, Layer3, and Layer4, respectively. Their channel dimensions are {64, 64, 128, 256, 512}, reflecting the gradual expansion of feature capacity along the encoder hierarchy. These feature maps provide complementary visual information across different abstraction levels. Specifically, shallow features ($F_{\text{1}},F_{\text{2}}$) retain fine-grained texture, edge, and boundary cues, whereas deeper features ($F_{\text{3}},F_{\text{4}},F_{\text{5}}$) encode increasingly compact semantic representations that provide progressively more abstract semantic representations for subsequent segmentation.

An important advantage of ResNet34 lies in its residual learning mechanism [32], which facilitates optimization stability and enables effective hierarchical representation learning. For polyp segmentation, this property is particularly beneficial because polyps often exhibit weak boundaries, irregular morphology, and subtle intensity variations relative to surrounding healthy mucosal tissues. Therefore, efficient and discriminative local feature extraction remains a critical prerequisite before introducing lightweight global context modeling.

In LiteMamba-Seg, the encoder is not intended to perform heavy global reasoning by itself. Instead, it provides a strong and computationally efficient convolutional feature backbone, upon which the subsequent Unified Mamba Blocks further enhance long-range contextual perception at the deepest bottleneck and the early decoder reconstruction stage.

### Unified Mamba block

To introduce efficient contextual modeling into the segmentation framework, LiteMamba-Seg employs a lightweight Mamba-based processing block. The motivation is that standard convolutional operations mainly model local neighborhoods, whereas Mamba provides an efficient sequence-based mechanism for capturing longer-range dependencies with linear complexity [11].

Given an input feature map $F\in R^{B\times C\times H\times W}$, the feature is first flattened along the spatial dimensions and rearranged into a one-dimensional token sequence:


$$\begin{array}{c} X=\text{Flatten}(F)\in R^{B\times (H\cdot W)\times C} \end{array}$$ (4)

Here, each spatial location is treated as an individual token, while the channel dimension C serves as the embedding dimension. To stabilize feature distribution before sequence modeling, layer normalization is applied:


$$\begin{array}{c} \hat{X}=LN(X) \end{array}$$ (5)

The normalized sequence is then processed by the Mamba operator:


$$\begin{array}{c} X^{\prime }=M\left(\hat{X}\right) \end{array}$$ (6)

where M(·) denotes the Mamba-based state-space sequence modeling function [11]. In principle, Mamba follows a selective state-space formulation in which input-dependent parameters control hidden-state transition and output generation, enabling efficient long-range dependency modeling with linear complexity.

After sequence modeling, the output sequence is reshaped back into the spatial feature form:


$$\begin{array}{c} F^{\prime }=\text{Reshape}\left(X^{\prime }\right)\in R^{B\times C\times H\times W} \end{array}$$ (7)

To improve spatial coherence and maintain compatibility with subsequent convolutional decoding stages, a lightweight convolutional refinement layer is applied:


$$\begin{array}{c} \tilde{F}=R\left(F^{\prime }\right) \end{array}$$ (8)

where R(·) denotes a 1 ×1 convolution followed by batch normalization and ReLU activation. Finally, a residual connection is introduced to preserve local structural information and facilitate optimization:


$$\begin{array}{c} F_{\text{out}}=F+\tilde{F} \end{array}$$ (9)

The resulting processing flow is summarized in Fig 2. As illustrated, the Unified Mamba Block combines sequence-based contextual modeling with lightweight convolutional refinement and residual preservation, allowing global dependency modeling to be incorporated without substantially increasing architectural complexity.

**Fig 2:**
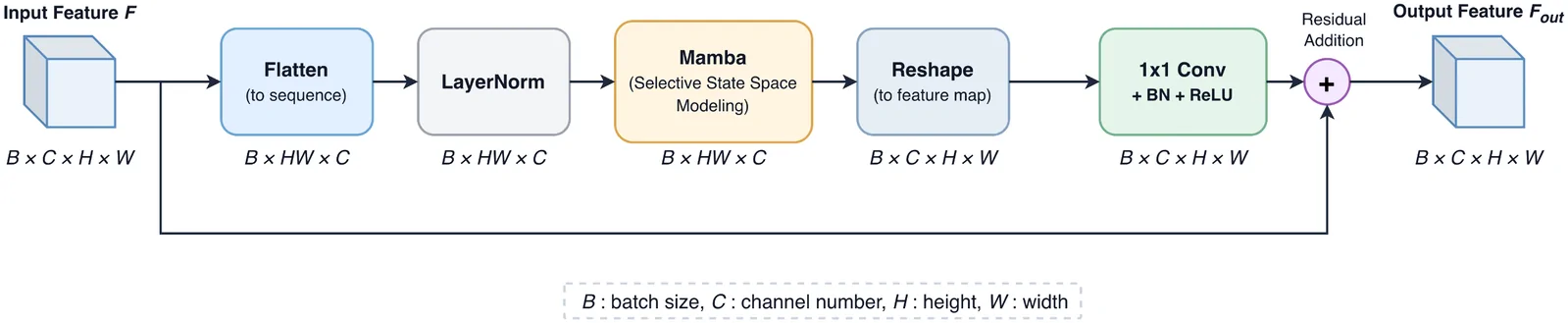
Structure of the Unified Mamba Block.

In the final LiteMamba-Seg architecture, the Unified Mamba Block is inserted at two locations. The first is the bottleneck stage, where it is applied to the deepest encoder feature $F_{\text{5}}$. The second is after the first decoder reconstruction stage ($D_{\text{4}}$), where the decoder feature is further processed before being passed to the next reconstruction stage. For ablation analysis, the same block is also evaluated at the Layer3 encoder stage to examine the effect of different placement strategies.

In this study, the Layer3 setting is considered only in the ablation analysis, whereas the final LiteMamba-Seg model retains the bottleneck and $D_{\text{4}}$ placements. This comparison is treated as an exploratory cross-stage analysis, with more closely matched placement comparisons conducted separately within the $D_{\text{4}}$ stage.

By combining sequence modeling with lightweight refinement and residual preservation, the Unified Mamba Block introduces contextual modeling into the network while keeping the overall design compact.

### Decoder design

The decoder of LiteMamba-Seg progressively reconstructs high-resolution segmentation predictions from the encoded feature representations. To keep the overall framework lightweight, a simple decoder topology is adopted instead of heavy multi-branch aggregation or densely stacked refinement modules. This design follows the general encoder–decoder principle of U-Net-like architectures, in which high-level semantic features are gradually upsampled and fused with the corresponding encoder features through skip connections [14].

Starting from the bottleneck output, the decoder performs spatial recovery through four successive reconstruction stages. Let $D_{\text{4}},D_{\text{3}},D_{\text{2}}$ and $D_{\text{1}}$ denote the decoder features from deep to shallow stages. In the first decoder stage, the bottleneck feature is first upsampled, concatenated with the corresponding encoder feature $F_{\text{4}}$, and then refined by the decoder convolution block:


$$\begin{array}{c} D_{\text{4}}=\phi_{\text{4}}\left(\text{Concat}\left(\text{Up}(B),F_{\text{4}}\right)\right) \end{array}$$ (10)

Where B denotes the bottleneck output, Up(·) denotes transposed convolution upsampling, Concat(·) denotes channel-wise concatenation, and $\phi_{\text{4}}(\cdot )$ denotes the convolutional refinement block at the $D_{\text{4}}$ stage. Transposed convolution was adopted to provide learnable spatial upsampling while preserving the standard stage-wise decoder structure and alignment with the corresponding skip features. In the main architecture, the fused $D_{\text{4}}$ feature is further processed by a Unified Mamba Block after the convolutional refinement at the first decoder stage. For the controlled ablation study, four additional insertion points were considered within the $D_{\text{4}}$ stage: before upsampling, before skip fusion, after fusion, and after convolutional refinement. These variants were introduced solely to isolate placement effects and are not part of the final LiteMamba-Seg architecture.

The remaining decoder stages follow the same progressive reconstruction pattern:


$$\begin{array}{c} D_{i}=\phi_{i}\left(\text{Concat}\left(\text{Up}\left(D_{i+\text{1}}\right),F_{i}\right)\right)\;,\;i\in \{\text{1},\text{2},\text{3}\} \end{array}$$ (11)

Accordingly, the $D_{\text{3}}$ stage fuses the upsampled $D_{\text{4}}$ feature with $F_{\text{3}}$, the $D_{\text{2}}$ stage fuses the upsampled $D_{\text{3}}$ feature with $F_{\text{2}}$, and the $D_{\text{1}}$ stage fuses the upsampled $D_{\text{2}}$ feature with $F_{\text{1}}$. Through this hierarchical recovery process, coarse semantic features from deep layers are gradually transformed into dense pixel-level representations.

Because the decoder produces hierarchical feature representations at multiple reconstruction stages, LiteMamba-Seg adopts a deep supervision strategy during training [33]. Specifically, lightweight prediction heads are applied to $D_{\text{1}},D_{\text{2}},D_{\text{3}}$, and $D_{\text{4}}$, generating four supervised predictions in total: one main high-resolution output and three auxiliary outputs. All outputs are upsampled to the input resolution for supervision during training, whereas only the highest-resolution prediction is retained during inference.

This progressive reconstruction strategy enables LiteMamba-Seg to combine hierarchical feature fusion with lightweight decoding while preserving a simple overall decoder structure.

### Efficiency-oriented design

A central design objective of LiteMamba-Seg is to introduce contextual modeling without substantially increasing computational cost. In practical colonoscopy scenarios, computational efficiency and inference speed are important considerations for practical deployment. Therefore, computational efficiency is considered as part of the architectural design rather than only as a post-hoc evaluation metric.

The lightweight design of LiteMamba-Seg is supported by three main choices. First, the framework adopts ResNet34 as the encoder backbone, which provides hierarchical convolutional feature extraction with moderate complexity [32]. Second, the decoder follows a simple reconstruction topology based on transposed convolutions, skip fusion, and local convolutional refinement, avoiding heavy multi-branch aggregation or densely stacked refinement modules [14]. Third, contextual modeling is introduced through selective Mamba integration rather than dense self-attention, so that longer-range dependency modeling can be incorporated with relatively limited additional overhead [11].

Compared with Transformer-based global modeling, whose self-attention mechanism scales quadratically with sequence length [20], the Mamba operator provides a more efficient alternative for sequence-based contextual modeling [11]. This property is especially relevant for dense prediction tasks such as medical image segmentation, where flattening high-resolution feature maps can lead to very long token sequences. In LiteMamba-Seg, the Unified Mamba Block is therefore inserted only at selected stages of the network rather than throughout the entire architecture.

Through these design choices, LiteMamba-Seg combines hierarchical convolutional representation learning with lightweight contextual modeling while keeping the overall framework compact. The actual trade-off between segmentation performance and computational efficiency is further examined in the experimental section.

## Experimental results and analysis

To evaluate LiteMamba-Seg from multiple perspectives, this chapter presents experiments covering the experimental setup, benchmark segmentation performance, computational efficiency, ablation analysis, multi-seed stability, robustness to input perturbations, prediction reliability, and qualitative results. We first describe the datasets, evaluation metrics, and implementation details, and then compare LiteMamba-Seg with representative segmentation methods under both in-domain and cross-dataset settings. This is followed by computational profiling and a series of ablation studies examining component contributions and Mamba placement strategies. Additional analyses are conducted to assess reproducibility across random seeds, robustness to input perturbations, boundary quality, calibration, and lesion-level false-positive behavior. Finally, qualitative examples are provided to complement the quantitative findings.

### Experimental setup

To evaluate the segmentation performance, computational efficiency, reproducibility, and robustness of LiteMamba-Seg, experiments were conducted on four publicly available polyp segmentation datasets. This section describes the datasets, evaluation metrics, training protocol, and implementation settings used throughout the experiments.

#### Datasets.

Following the benchmark partition widely adopted in PraNet-style polyp segmentation studies [16], experiments were conducted on four public datasets: Kvasir-SEG [34], CVC-ClinicDB [35], CVC-ColonDB [36], and ETIS-LaribPolypDB [37]. The original development pool contained 1,450 image-mask pairs, including 900 images from Kvasir-SEG and 550 images from CVC-ClinicDB. To enable model selection without using the predefined test subsets, the 1,450 development samples were first sorted by image identifier to establish a deterministic initial ordering and then randomly shuffled using a fixed split seed of 42 before applying a 90:10 training-validation split. This resulted in 1,305 image-mask pairs for model optimization and 145 pairs for internal validation. The resulting split was fixed before any data augmentation and remained unchanged across all model configurations and multi-seed runs. Only the training subset was subjected to stochastic data augmentation, whereas the validation subset underwent deterministic resizing and normalization.

For evaluation, the predefined test subsets contained 100 images from Kvasir-SEG, 62 images from CVC-ClinicDB, 380 images from CVC-ColonDB, and 196 images from ETIS-LaribPolypDB. The Kvasir-SEG and CVC-ClinicDB test subsets were disjoint from the development pool at the image level and were reserved for final in-domain evaluation. CVC-ColonDB and ETIS-LaribPolypDB were used for cross-dataset evaluation without fine-tuning. None of the test subsets were used for model training, gradient updates, checkpoint selection, or hyperparameter tuning. Exact training and validation image identifiers are provided in the accompanying code repository to facilitate reproducibility.

All datasets used in this study are publicly available and de-identified. No new human participants were recruited, and the authors did not have access to any identifiable patient information. Therefore, no additional institutional ethics approval or informed consent was required for this secondary analysis of publicly available data.

#### Evaluation metrics.

Segmentation performance was evaluated using four standard pixel-level metrics: Dice coefficient, Intersection over Union (IoU), Recall, and Precision. Dice and IoU quantify the overlap between predicted masks and ground-truth annotations, while Recall and Precision characterize foreground sensitivity and positive predictive performance, respectively.

For each test image, the raw model output was first resized to the original image resolution using bilinear interpolation with align_corners = False, converted to a probability map using the sigmoid function, and binarized using a fixed threshold of 0.5. The corresponding ground-truth mask was also binarized using a threshold of 0.5. Dice, IoU, Recall, and Precision were computed independently for each image and then macro-averaged over all images in each test dataset. A smoothing constant of $\text{1}\times {\text{1}\text{0}}^{-\text{5}}$ was applied to the Dice and IoU calculations to avoid undefined zero-denominator cases. Precision and Recall used explicit zero-denominator handling rather than smoothing. When no foreground pixels were predicted for an image containing foreground, Precision was defined as 0; when both prediction and ground truth contained no foreground, Precision and Recall were defined as 1. No empty ground-truth masks were present in the evaluated test sets, so the latter case did not affect the reported results. No connected-component filtering, morphological processing, or other post-processing was applied.

To complement overlap-based evaluation, boundary quality was assessed using Boundary IoU and the 95th-percentile Hausdorff distance (HD95). Prediction calibration was evaluated using expected calibration error (ECE) with 15 equally spaced confidence bins, while lesion-level false-positive behavior was measured as the average number of false-positive connected components per image (FP/image). These additional metrics were computed from the clean-image predictions.

Robustness to image degradation was further evaluated using five perturbations applied independently to the test images: Gaussian blur with a radius of 2.0, JPEG compression with a quality factor of 50, brightness enhancement by a factor of 1.2, contrast reduction by a factor of 0.8, and low-resolution degradation. For the low-resolution condition, each image was downsampled to 176 × 176 and subsequently resized back to its original spatial resolution using bilinear interpolation before the standard 35 × 352 preprocessing. No retraining or fine-tuning was performed for any perturbed condition.

Computational efficiency was characterized using the number of trainable parameters (Params), floating-point operations (GFLOPs), model-level inference latency, and frames per second (FPS).

#### Implementation details.

All experiments were implemented in PyTorch [38]. Input colonoscopy images and corresponding masks were uniformly resized to 352 × 352. The encoder was initialized with ImageNet-pretrained ResNet-34 weights unless otherwise specified in a controlled experiment. LiteMamba-Seg was optimized using AdamW [39] with an initial learning rate of $\text{1}\times {\text{1}\text{0}}^{-\text{4}}$ and a weight decay of $\text{1}\times {\text{1}\text{0}}^{-\text{4}}$. A cosine annealing learning-rate scheduler [40] was applied over 100 training epochs with $T_{max}=\text{1}\text{0}\text{0}$ a minimum learning rate of $\text{1}\times {\text{1}\text{0}}^{-\text{6}}$. The batch size was set to 16, and mixed-precision training was enabled.

To improve training diversity and reduce overfitting, online data augmentation was applied using the Albumentations library [41], including random horizontal and vertical flipping, random 90-degree rotations, shift-scale-rotate transformations, elastic and grid distortions, intensity perturbations, blur-based augmentation, and coarse dropout. Coarse dropout was applied to the input image while preserving the corresponding segmentation mask. Only the training subset was subjected to stochastic augmentation, whereas the internal validation subset and test datasets underwent deterministic resizing and ImageNet normalization.

The overall optimization objective combined StructureLoss with Boundary IoU loss and deep supervision from four prediction outputs, consisting of one main output and three auxiliary outputs. The main output was supervised using StructureLoss together with a weighted Boundary IoU loss, while the auxiliary outputs were supervised using StructureLoss with weights of 0.3, 0.2, and 0.1, respectively. Boundary IoU loss was computed independently for each image and averaged across the mini-batch, with the boundary-loss computation performed in FP32 to improve numerical stability under mixed-precision training. The best checkpoint was selected according to the highest macro-averaged Dice score on the fixed internal validation subset.

For the multi-seed analysis, the final LiteMamba-Seg configuration was independently trained using five random seeds: 42, 3407, 2025, 666, and 1234. All training and inference experiments were conducted on an NVIDIA GeForce RTX 3080 Ti GPU with 12 GB VRAM under a CUDA-enabled PyTorch environment. Model-level inference speed was measured using a batch size of 1 with 100 warm-up iterations followed by 300 timed iterations.

### Quantitative results on benchmark datasets

To evaluate the overall segmentation performance of LiteMamba-Seg, we compare the proposed framework with several representative methods on four widely used polyp segmentation benchmarks. The evaluation encompasses the in-domain test sets of Kvasir-SEG and CVC-ClinicDB, together with CVC-ColonDB and ETIS-LaribPolypDB under the cross-dataset evaluation setting. This comparison aims to examine whether selective Mamba integration can maintain competitive segmentation performance across both settings while preserving a compact architectural design.

Table 1 summarizes the quantitative comparison in terms of Dice score. For the primary benchmark comparison, we report the final LiteMamba-Seg configuration trained with seed 2025, while the multi-seed stability of the same configuration is further examined in the Multi-Seed Stability Analysis section. LiteMamba-Seg achieves Dice scores of 0.916 on Kvasir-SEG and 0.927 on CVC-ClinicDB. These results indicate that LiteMamba-Seg achieves strong in-domain segmentation performance with selective Mamba integration.

**Table 1:** Quantitative comparison of Dice scores on four benchmark datasets.

Method	Kvasir ↑	ClinicDB ↑	ColonDB ↑	ETIS ↑
PraNet [16]	0.898	0.899	0.712	0.628
TransFuse [22]	0.905	0.898	0.750	0.673
Polyp-PVT [23]	0.917	0.937	0.808	0.787
VM-UNet [28]	0.891	0.901	0.712	0.798
VM-UNet V2 [42]	0.914	0.944	0.758	0.839
**LiteMamba-Seg (Ours)**	**0.916**	**0.927**	**0.785**	**0.766**

Note: Results of the compared methods are taken from their corresponding original publications for reference and may differ in training splits, preprocessing pipelines, implementation details, and evaluation settings. LiteMamba-Seg results are obtained using the final model trained with seed 2025 under the experimental protocol described in the Experimental Setup section.

Under the cross-dataset evaluation setting, LiteMamba-Seg achieves Dice scores of 0.785 on CVC-ColonDB and 0.766 on ETIS-LaribPolypDB without fine-tuning. These results indicate that the proposed compact architecture retains useful cross-dataset generalization without fine-tuning.

The subsequent experiments further examine computational efficiency, architectural placement, multi-seed stability, and robustness-related behavior under the same experimental protocol.

### Computational efficiency

In addition to segmentation accuracy, computational efficiency is an important consideration for polyp segmentation in dynamic endoscopic scenarios, where low-latency inference is desirable. To characterize the efficiency of LiteMamba-Seg, we evaluate the model in terms of trainable parameter count, estimated computational complexity (Est. GFLOPs), model-level inference latency, and inference speed measured in frames per second (FPS).

As shown in Table 2, under an input resolution of 352 × 352, LiteMamba-Seg contains 26.81 M trainable parameters and requires 13.89 estimated GFLOPs. On an NVIDIA GeForce RTX 3080 Ti GPU, the model achieves an average inference speed of 166.73 FPS, corresponding to an average model-level latency of 6.00 ms per image. These measurements characterize the computational profile of the final LiteMamba-Seg configuration under the specified experimental environment.

**Table 2:** Computational efficiency of LiteMamba-Seg.

Method	Params (M) ↓	GFLOPs (G) ↓	Latency (ms) ↓	FPS ↑
**LiteMamba-Seg (Ours)**	26.81	13.89	6.00	166.73

Because inference speed is strongly affected by hardware, input resolution, and implementation details, cross-study FPS values are not used here for direct efficiency comparison. Instead, the following ablation analyses provide same-environment measurements for the internally evaluated configurations, allowing the computational effect of different Mamba integration strategies to be examined under a consistent experimental setting.

### Ablation study

To better understand how the final LiteMamba-Seg configuration is formed, we conduct a series of ablation experiments focusing on three aspects: preliminary component analysis, cross-stage Mamba placement, and controlled placement within the $D_{\text{4}}$ decoder stage. All compared configurations follow the same training-validation split and evaluation protocol. The purpose of these experiments is to examine how individual design choices affect segmentation performance and to determine whether the effectiveness of Mamba integration depends on where the sequence-modeling block is introduced.

#### Preliminary component analysis.

Before investigating Mamba placement, we first examine the contributions of local convolutional refinement and bottleneck Mamba integration. Four configurations are compared: the plain baseline, convolutional refinement only, bottleneck Mamba only, and their combination. This analysis establishes the base configuration for the subsequent placement experiments.

As shown in Table 3, individually adding convolutional refinement or bottleneck Mamba improves the Dice score across all four datasets compared with the baseline. Convolutional refinement achieves Dice scores of 0.911 on Kvasir-SEG, 0.909 on CVC-ClinicDB, 0.756 on CVC-ColonDB, and 0.710 on ETIS-LaribPolypDB. Similarly, bottleneck Mamba achieves 0.915, 0.918, 0.773, and 0.705, respectively. These results indicate that both local refinement and bottleneck sequence modeling contribute to segmentation performance, although their relative effects vary across datasets.

**Table 3:** Effect of convolutional refinement and bottleneck Mamba integration in terms of Dice.

Variant	Bottleneck	Conv. Refine	Kvasir ↑	ClinicDB ↑	ColonDB ↑	ETIS ↑
**Baseline**	✗	✗	0.908	0.883	0.745	0.690
**+ Conv. Refine**	✗	✓	0.911	0.909	0.756	0.710
**+ Bottleneck**	✓	✗	0.915	0.918	0.773	0.705
**+ Conv. Refine + Bottleneck**	✓	✓	0.910	0.919	0.775	0.738

When convolutional refinement and bottleneck Mamba are combined, the resulting configuration achieves Dice scores of 0.910 on Kvasir-SEG, 0.919 on CVC-ClinicDB, 0.775 on CVC-ColonDB, and 0.738 on ETIS-LaribPolypDB. Although this combination does not produce the highest Kvasir-SEG score among the preliminary variants, it provides the strongest results on CVC-ClinicDB, CVC-ColonDB, and ETIS-LaribPolypDB, with the improvement being particularly evident on ETIS-LaribPolypDB. The combined configuration therefore provides a suitable base for investigating whether an additional Mamba block can further improve segmentation performance.

Taken together, the preliminary analysis shows that convolutional refinement and bottleneck Mamba integration provide complementary but dataset-dependent contributions. Based on the overall performance across the four benchmarks, the configuration combining convolutional refinement with bottleneck Mamba integration is adopted as the common base for the subsequent Mamba placement analysis.

#### Cross-stage Mamba placement analysis.

Starting from the Conv. Refine + Bottleneck configuration, we next investigate the placement of an additional Mamba block. Two representative locations are considered: Layer3 on the encoder side and $D_{\text{4}}\;$ on the decoder side. This experiment provides an initial cross-stage comparison of intermediate encoder modeling and decoder-side Mamba integration.

As shown in Table 4, inserting the additional Mamba block at Layer3 reduces Dice performance relative to the common base configuration across all four datasets. The Dice scores decrease from 0.910 to 0.907 on Kvasir-SEG, from 0.919 to 0.908 on CVC-ClinicDB, from 0.775 to 0.761 on CVC-ColonDB, and from 0.738 to 0.717 on ETIS-LaribPolypDB. The Layer3 configuration also reduces inference speed from 185.08 FPS to 165.41 FPS. These results indicate that the additional encoder-side Mamba block does not provide a favorable accuracy-efficiency trade-off under the investigated setting.

**Table 4:** Effect of second-stage Mamba placement under the Conv. Refine + Bottleneck setting.

Variant	Layer3	D4	Kvasir ↑	ClinicDB ↑	ColonDB ↑	ETIS ↑	FPS ↑
**Conv. Refine + Bottleneck**	✗	✗	0.910	0.919	0.775	0.738	185.08
**+ Layer3**	✓	✗	0.907	0.908	0.761	0.717	165.41
**+ D4**	✗	✓	0.916	0.927	0.785	0.766	166.73

A different pattern is observed for decoder-side $D_{\text{4}}$ integration. Relative to the same Conv. Refine + Bottleneck configuration, adding Mamba at $D_{\text{4}}$ increases the Dice score from 0.910 to 0.916 on Kvasir-SEG, from 0.919 to 0.927 on CVC-ClinicDB, from 0.775 to 0.785 on CVC-ColonDB, and from 0.738 to 0.766 on ETIS-LaribPolypDB. The improvement is most pronounced on ETIS-LaribPolypDB, while the model retains an inference speed of 166.73 FPS. Within this exploratory cross-stage comparison, the decoder-side placement therefore provides a more favorable performance profile than the Layer3 placement.

However, Layer3 and $D_{\text{4}}$ operate at different spatial resolutions and feature dimensions. Consequently, the comparison in Table 4 cannot fully isolate the effect of placement from differences in representation scale and computational characteristics. We therefore conduct an additional controlled experiment within the $D_{\text{4}}$ stage to examine the effect of Mamba insertion position under more closely matched conditions.

#### Controlled Mamba placement within the $D_{4}$ stage.

To further isolate the effect of Mamba placement, a controlled $D_{\text{4}}$ stage architecture was constructed in which the same 256-dimensional Mamba block was inserted at four locations: before upsampling (Pre-up), before skip-feature fusion (Pre-fusion), after fusion (Post-fusion), and after decoder convolution (Post-conv). A configuration without the additional $D_{\text{4}}$ Mamba block (None) was included as a reference. All variants used the same data split, training protocol, and validation-based checkpoint-selection procedure.

The Pre-fusion, Post-fusion, and Post-conv variants operate at the same 22 × 22 spatial resolution and have identical parameter counts and computational complexity (26.22 M parameters and 13.56 GFLOPs). The Pre-up variant uses the same Mamba dimensionality and parameter count but operates at the lower 11 × 11 resolution, resulting in a slightly lower computational cost of 13.54 GFLOPs. The None configuration contains no additional $D_{\text{4}}$ Mamba block and therefore has fewer parameters.

Among the controlled variants, the Post-conv placement achieves the highest Dice score on all four test datasets, reaching 0.918 on Kvasir-SEG, 0.922 on CVC-ClinicDB, 0.791 on CVC-ColonDB, and 0.754 on ETIS-LaribPolypDB. In contrast, placing the same Mamba block immediately before or after feature fusion does not provide a comparable improvement. This result suggests that the benefit observed for decoder-side Mamba integration is not explained by skip-feature fusion alone; within the controlled $D_{\text{4}}$ setting, applying Mamba after local decoder convolution yields the most favorable segmentation performance.

The controlled architecture in Table 5 was introduced specifically to isolate placement effects and is not identical to the final LiteMamba-Seg decoder used in Tables 1 and 2. Therefore, its parameter count and GFLOPs should not be interpreted as the complexity of the final model. Rather, the experiment provides complementary evidence that the relative location of Mamba within the decoder stage can materially affect segmentation behavior. Together with the cross-stage results in Table 4, these findings support the use of decoder-side post-convolution Mamba integration in the final LiteMamba-Seg configuration.

**Table 5:** Controlled analysis of Mamba placement within the D4 decoder stage.

Position	Params (M) ↓	GFLOPs (G) ↓	Kvasir ↑	ClinicDB ↑	ColonDB ↑	ETIS ↑
**None**	25.71	13.53	0.912	0.919	0.778	0.724
**Pre-up**	26.22	13.54	0.915	0.911	0.763	0.727
**Pre-fusion**	26.22	13.56	0.913	0.910	0.780	0.724
**Post-fusion**	26.22	13.56	0.910	0.900	0.768	0.723
**Post-conv**	**26.22**	**13.56**	**0.918**	**0.922**	**0.791**	**0.754**

### Multi-seed stability analysis

To further assess the reproducibility of the final LiteMamba-Seg configuration, we conducted independent experiments using five random seeds (42, 3407, 2025, 666, and 1234). The final configuration includes convolutional refinement, a bottleneck Mamba block, and a $D_{\text{4}}$ Mamba block. All runs used the same training-validation split and experimental protocol, with only the training seed varied. This analysis was conducted to examine the sensitivity of the reported performance to random initialization.

Table 6 reports the individual results obtained from each of the five random seeds, together with the corresponding mean ± standard deviation and 95% confidence intervals for Dice, IoU, Recall, and Precision. On the two in-domain datasets, LiteMamba-Seg shows relatively limited variation across runs, with mean Dice scores of 0.915 ± 0.005 on Kvasir-SEG and 0.918 ± 0.006 on CVC-ClinicDB. Larger variability is observed under cross-dataset evaluation, where the mean Dice scores are 0.765 ± 0.014 on CVC-ColonDB and 0.744 ± 0.020 on ETIS-LaribPolypDB. The corresponding 95% confidence intervals are [0.909, 0.921], [0.911, 0.926], [0.748, 0.782], and [0.719, 0.768], respectively. These results indicate that the final configuration is less sensitive to training randomness on the in-domain datasets, whereas greater run-to-run variability is observed under the cross-dataset evaluation setting.

**Table 6:** Performance of LiteMamba-Seg across five random seeds.

Dataset	Seed/ Summary	Dice ↑	IoU ↑	Recall ↑	Precision ↑
**Kvasir**	42	0.920	0.872	0.935	0.925
	3407	0.910	0.859	0.928	0.917
	2025	0.916	0.868	0.925	0.927
	666	0.918	0.868	0.924	0.930
	1234	0.910	0.860	0.920	0.928
	**Mean ± SD**	**0.915 ± 0.005**	**0.865 ± 0.006**	**0.926 ± 0.006**	**0.925 ± 0.006**
	**95% CI**	**[0.909, 0.921]**	**[0.858, 0.873]**	**[0.920, 0.933]**	**[0.919, 0.932]**
**ClinicDB**	42	0.915	0.866	0.955	0.899
	3407	0.913	0.861	0.955	0.893
	2025	0.927	0.875	0.962	0.907
	666	0.922	0.869	0.966	0.897
	1234	0.914	0.864	0.951	0.906
	**Mean ± SD**	**0.918 ± 0.006**	**0.867 ± 0.006**	**0.958 ± 0.007**	**0.901 ± 0.006**
	**95% CI**	**[0.911, 0.926]**	**[0.861, 0.874]**	**[0.950, 0.965]**	**[0.894, 0.908]**
**ColonDB**	42	0.771	0.692	0.856	0.779
	3407	0.764	0.684	0.844	0.777
	2025	0.785	0.705	0.833	0.807
	666	0.752	0.670	0.846	0.761
	1234	0.752	0.682	0.804	0.771
	**Mean ± SD**	**0.765 ± 0.014**	**0.687 ± 0.013**	**0.837 ± 0.020**	**0.779 ± 0.017**
	**95% CI**	**[0.748, 0.782]**	**[0.671, 0.703]**	**[0.812, 0.861]**	**[0.758, 0.800]**
**ETIS**	42	0.753	0.661	0.902	0.690
	3407	0.722	0.634	0.867	0.669
	2025	0.766	0.684	0.871	0.727
	666	0.724	0.634	0.892	0.667
	1234	0.753	0.665	0.858	0.705
	**Mean ± SD**	**0.744 ± 0.020**	**0.655 ± 0.022**	**0.878 ± 0.019**	**0.692 ± 0.026**
	**95% CI**	**[0.719, 0.768]**	**[0.629, 0.682]**	**[0.855, 0.901]**	**[0.660, 0.723]**

Similar patterns are observed for IoU, Recall, and Precision. The standard deviations remain relatively small on Kvasir-SEG and CVC-ClinicDB, whereas CVC-ColonDB and ETIS-LaribPolypDB show greater variability, particularly in Recall and Precision. Among the four datasets, ETIS-LaribPolypDB exhibits the largest variation in Dice and Precision, suggesting greater sensitivity to training randomness under the cross-dataset setting. Nevertheless, the five-run results remain within a comparable performance range and provide a more representative estimate of the final model than a single-seed evaluation.

Seed 666 achieved the highest validation Dice (0.9442) among the five runs. However, the same seed did not consistently give the best test performance across datasets, indicating some run-to-run variation in generalization. As shown in Fig 3, the validation Dice curves exhibit broadly similar convergence trends across the five seeds. Although some fluctuations are observed during training, the overall optimization trajectories remain comparable. These results indicate that the final LiteMamba-Seg configuration shows generally reproducible training behavior, while greater variability is observed under cross-dataset evaluation.

**Fig 3:**
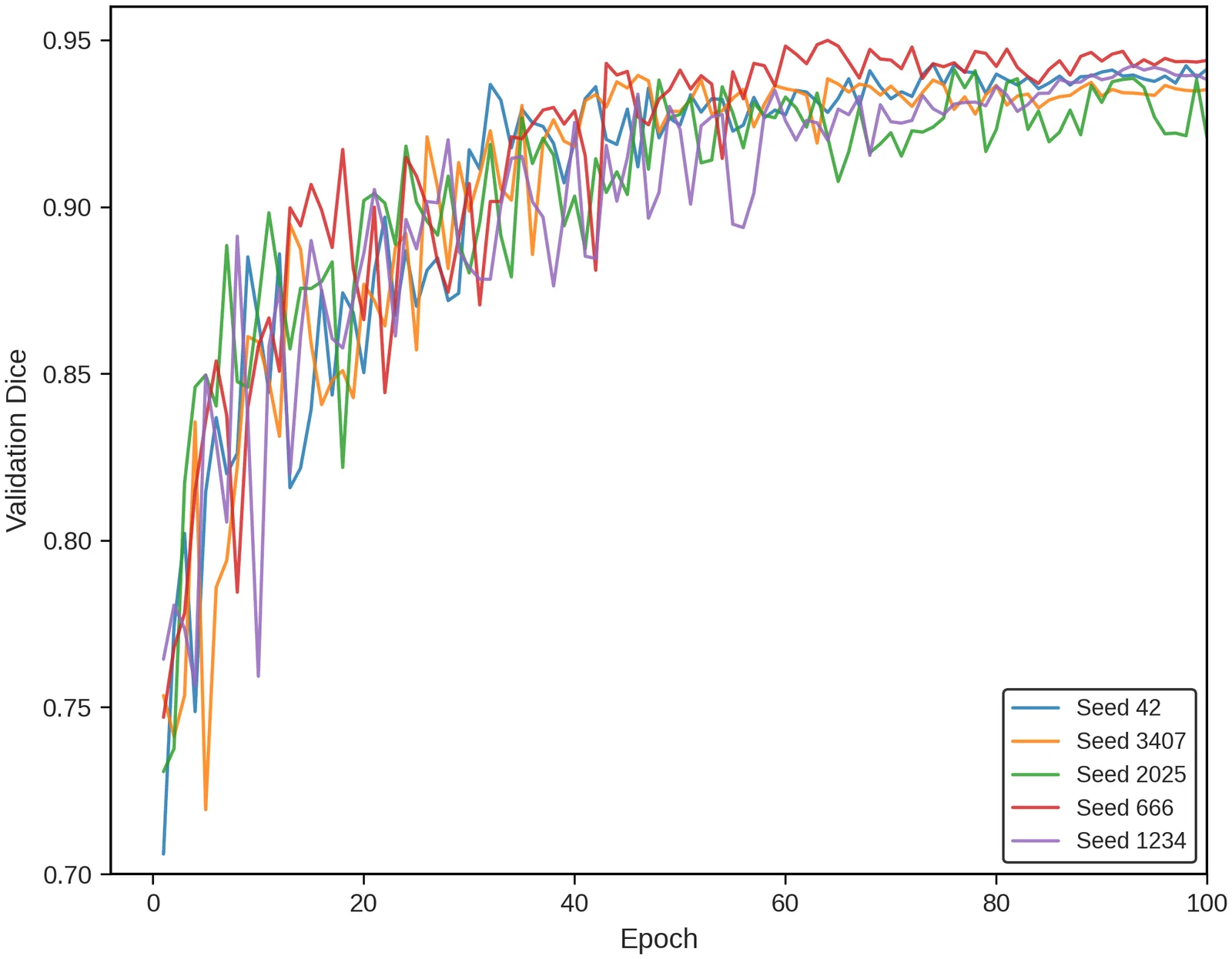
Validation Dice curves of the final LiteMamba-Seg configuration under five different random seeds.

### Robustness analysis

To further evaluate the behavior of LiteMamba-Seg beyond standard clean-image testing, additional analyses were conducted from two perspectives: robustness to common image perturbations and prediction reliability. The final LiteMamba-Seg model was compared with the non-Mamba baseline under the same evaluation protocol.

#### Input perturbation analysis.

To assess robustness to input degradation, five perturbations were independently applied to the test images, including Gaussian blur, JPEG compression, brightness variation, contrast variation, and resolution degradation. Gaussian blur was applied with a radius of 2.0, JPEG compression with a quality factor of 50, brightness was increased by a factor of 1.2, and contrast was reduced by a factor of 0.8. For the low-resolution condition, each image was first downsampled to 176 × 176 and then resized back to its original spatial resolution using bilinear interpolation before the standard 352 × 352 model preprocessing was applied. No retraining or fine-tuning was performed under any perturbed condition. Table 7 reports the Dice scores obtained on the clean and perturbed images.

**Table 7:** Dice performance under different input perturbations. Ours denotes LiteMamba-Seg.

Perturbation	Kvasir		ClinicDB		ColonDB		ETIS	
	Baseline	Ours	Baseline	Ours	Baseline	Ours	Baseline	Ours
**Clean**	0.908	**0.916**	0.883	**0.927**	0.745	**0.785**	0.690	**0.766**
**Blur**	0.911	**0.924**	0.852	**0.906**	0.734	**0.782**	0.673	**0.758**
**JPEG**	0.902	**0.916**	0.877	**0.894**	0.737	**0.783**	0.671	**0.765**
**Brightness**	0.906	**0.919**	0.893	**0.923**	0.755	**0.792**	0.662	**0.751**
**Contrast**	0.893	**0.918**	0.890	**0.918**	0.729	**0.786**	0.667	**0.760**
**Low-res**	0.912	**0.922**	0.883	**0.924**	0.739	**0.780**	0.637	**0.725**

As shown in Table 7, LiteMamba-Seg maintains higher Dice scores than the non-Mamba baseline under all evaluated perturbation conditions across the four datasets. On Kvasir-SEG, the Dice score remains close to or slightly above the clean-image result under several perturbations. On CVC-ClinicDB and CVC-ColonDB, LiteMamba-Seg also retains relatively stable performance across blur, compression, illumination changes, and resolution degradation.

The effect of degradation is more pronounced on ETIS-LaribPolypDB, particularly under the low-resolution condition, where the Dice score decreases from 0.766 on clean images to 0.725. Nevertheless, the corresponding baseline decreases from 0.690 to 0.637, and LiteMamba-Seg maintains higher Dice scores under all evaluated perturbations. These results indicate that the performance advantage of LiteMamba-Seg over the non-Mamba baseline is preserved under the considered image degradations, although the magnitude of performance change varies across datasets.

#### Boundary and reliability analysis.

Because overlap-based metrics alone do not fully characterize segmentation quality, additional evaluations were conducted using Boundary IoU, the 95th-percentile Hausdorff distance (HD95), expected calibration error (ECE), and lesion-level false-positive components per image. Boundary IoU and HD95 characterize boundary agreement, whereas ECE measures the agreement between predicted probabilities and observed pixel-level outcomes. FP/image quantifies the average number of false-positive connected components detected per image.

As shown in Table 8, LiteMamba-Seg achieves higher Boundary IoU and lower HD95 than the non-Mamba baseline on all four datasets. The differences are particularly pronounced on CVC-ColonDB and ETIS-LaribPolypDB, where the reductions in HD95 indicate improved agreement between the predicted and reference boundaries.

**Table 8:** Boundary quality and prediction reliability of the non-Mamba baseline and LiteMamba-Seg.

Metric	Kvasir ↑		ClinicDB ↑		ColonDB ↑		ETIS ↑	
	Baseline	Ours	Baseline	Ours	Baseline	Ours	Baseline	Ours
**Boundary IoU ↑**	0.649	**0.668**	0.664	**0.716**	0.486	**0.516**	0.451	**0.546**
**HD95 ↓**	47.77	**44.56**	29.55	**16.60**	100.84	**65.22**	233.43	**155.71**
**ECE ↓**	0.028	**0.022**	0.014	**0.007**	0.040	**0.036**	0.039	**0.016**
**FP/image ↓**	0.460	**0.330**	0.291	**0.259**	0.429	**0.385**	0.965	**0.455**

LiteMamba-Seg also yields lower ECE values across all datasets, indicating better calibration of the predicted probability maps under the adopted evaluation protocol. In addition, the number of lesion-level false-positive components is reduced on all four datasets. The largest reduction is observed on ETIS-LaribPolypDB, where FP/image decreases from 0.965 for the baseline to 0.455 for LiteMamba-Seg.

Taken together, the additional robustness, boundary, calibration, and false-positive analyses provide complementary evidence beyond Dice and IoU. The results show that the gains obtained by LiteMamba-Seg are accompanied by improved boundary quality and prediction reliability, while its performance advantage over the non-Mamba baseline is maintained under the evaluated input perturbations.

### Qualitative results

To provide a more intuitive comparison of segmentation behavior, we further visualize representative predictions on benchmark datasets as well as challenging cross-dataset cases. As shown in Fig 4, the error-overlay visualization allows the differences among methods to be directly observed in representative examples. On the in-domain cases from Kvasir-SEG and CVC-ClinicDB, LiteMamba-Seg produces predictions that are visually well aligned with the ground truth and, in several cases, shows fewer obvious missed regions or boundary deviations. On the cross-dataset cases from CVC-ColonDB and ETIS-LaribPolypDB, some compared methods exhibit incomplete responses or scattered false-positive regions, whereas LiteMamba-Seg often produces more spatially coherent predictions in the illustrated examples. These visual observations are broadly consistent with the quantitative results in Table 1, where LiteMamba-Seg shows competitive performance, although it does not outperform all compared methods on every benchmark.

**Fig 4:**
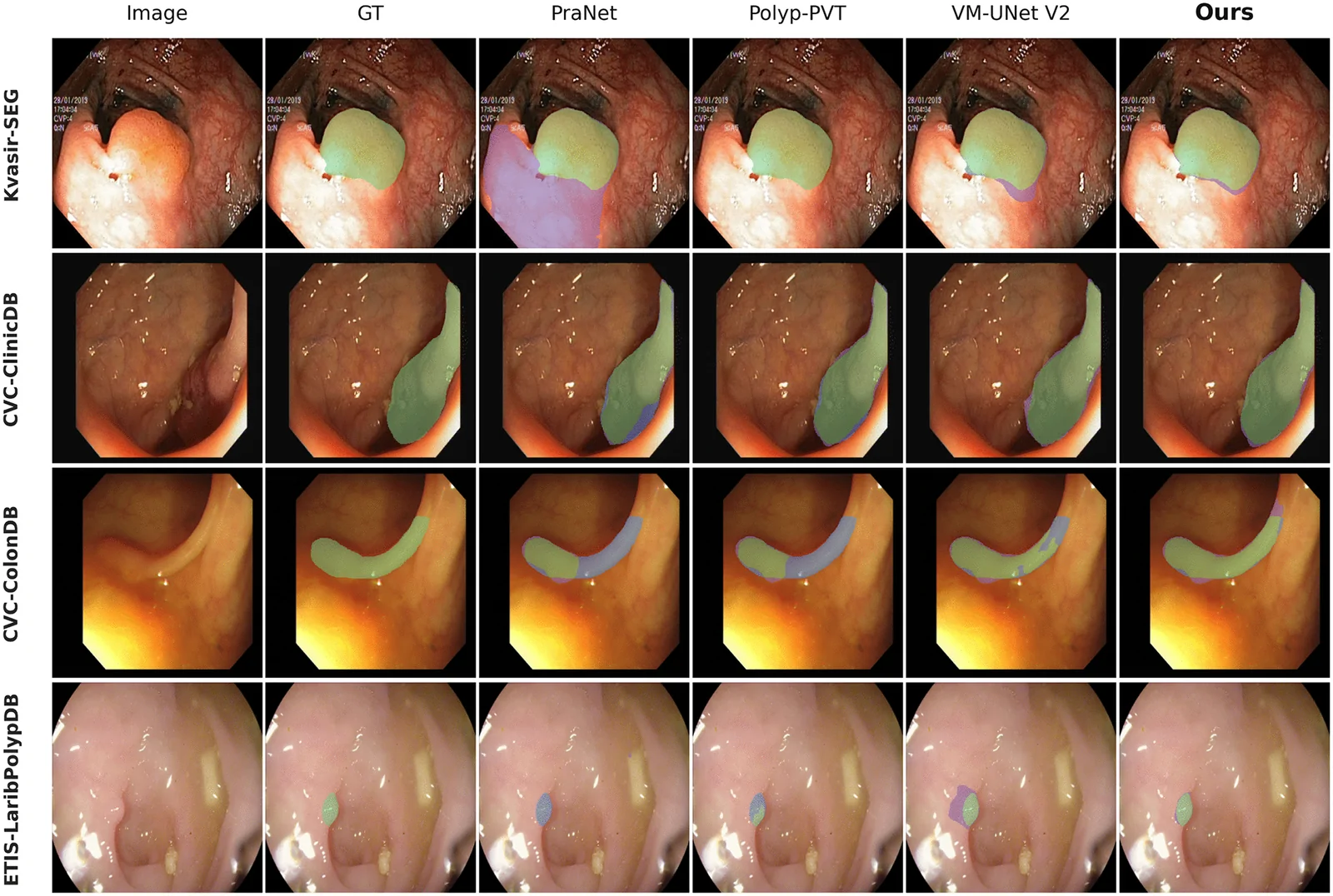
Qualitative comparison of segmentation results on representative samples from four benchmark datasets. In the error-overlay visualization, green, blue, and purple denote correctly predicted (TP), false-negative (FN), and false-positive (FP) regions, respectively.

Fig 5 further illustrates the effect of different Mamba integration stages. Compared with the plain baseline, the bottleneck Mamba configuration produces more complete lesion responses in some of the illustrated examples. However, when an additional Mamba block is inserted at Layer3, the resulting predictions do not consistently improve and, in several cases, exhibit weaker or less complete structural recovery. In contrast, replacing the Layer3 placement with decoder-side $D_{\text{4}}$ integration produces predictions that are more complete and more closely aligned with the ground truth in the selected cases. This qualitative trend is consistent with the quantitative ablation results in the cross-stage Mamba placement analysis, where $D_{\text{4}}$ placement shows more favorable performance than Layer3 placement.

**Fig 5:**
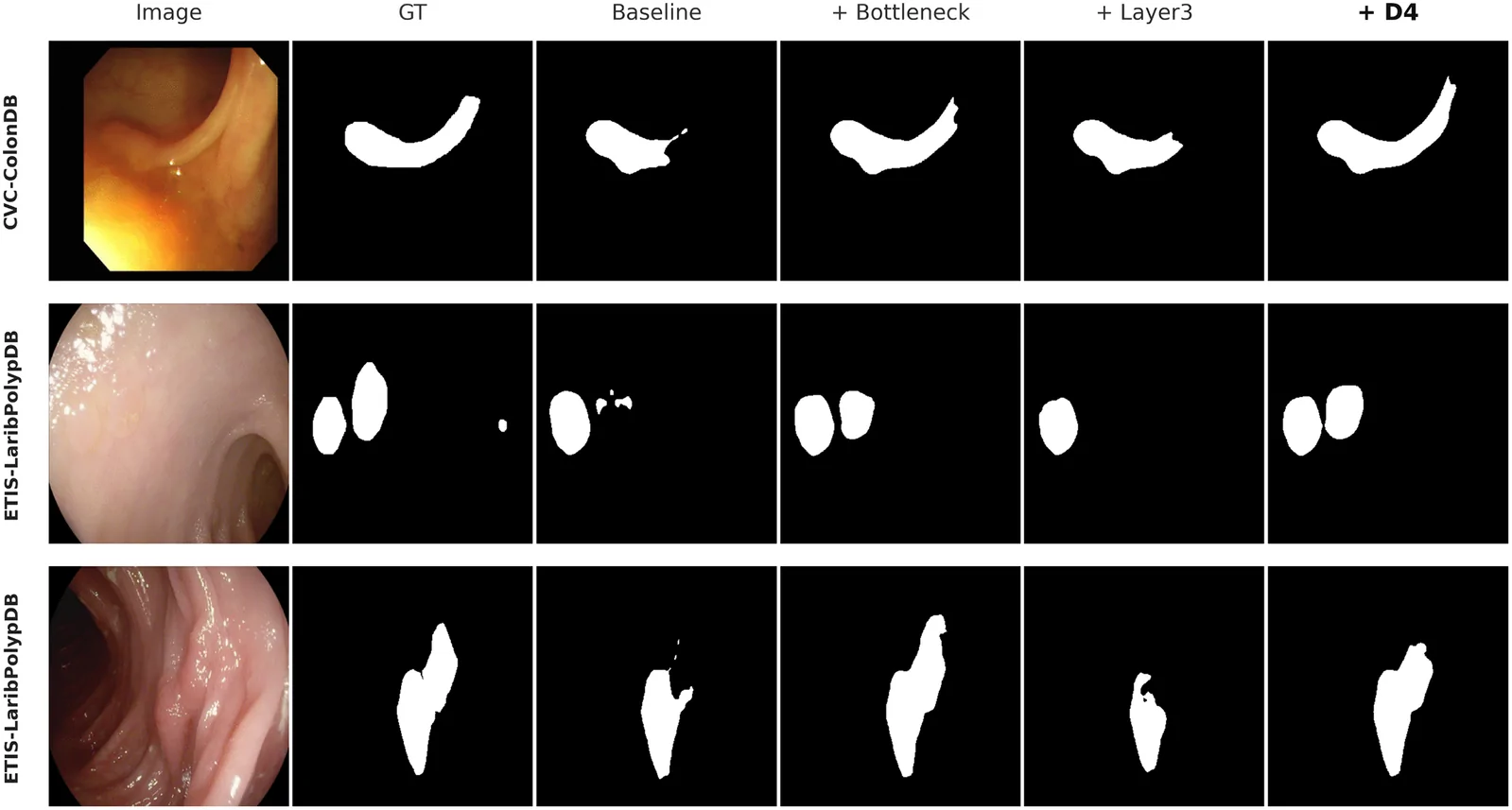
Qualitative ablation of Mamba integration stages on challenging external cases.

Taken together, Figs 4 and 5 suggest that the advantage of LiteMamba-Seg does not arise simply from adding more sequence modeling blocks, nor does it imply uniform superiority across all benchmarks. Rather, the visual evidence indicates that performance is strongly influenced by how the Mamba blocks are integrated within the network. In particular, replacing the encoder-side Layer3 placement with decoder-side $D_{\text{4}}$ integration appears to provide a more suitable complement to bottleneck modeling in the illustrated cases.

## Conclusion

In this study, we presented LiteMamba-Seg, a lightweight segmentation framework for polyp segmentation, and empirically evaluated how selected Mamba integration strategies influence segmentation performance under both in-domain and cross-dataset settings. Rather than increasing architectural complexity through extensive sequence modeling, the proposed framework focused on lightweight integration guided by placement strategy within a U-Net-like encoder–decoder architecture.

The experimental results show that bottleneck Mamba integration improves segmentation performance across the evaluated benchmarks, while the subsequent placement analysis indicates that the effect of an additional Mamba block varies with its integration stage. Introducing an additional Mamba block at the encoder-side Layer3 stage does not consistently improve performance, whereas decoder-side $D_{\text{4}}$ integration provides better quantitative performance than the Layer3 alternative across the evaluated benchmarks, with the qualitative examples showing a similar trend. The controlled $D_{\text{4}}$ placement experiments further show that post-convolution integration performs better than the other examined positions under closely matched model capacity.

LiteMamba-Seg achieves a favorable balance between segmentation accuracy and model-level inference efficiency while maintaining competitive performance across the evaluated benchmark datasets. The multi-seed experiments further demonstrate reproducible training behavior, while the perturbation, boundary, calibration, and false-positive analyses provide additional evidence of stable and reliable segmentation performance under the evaluated conditions. Although LiteMamba-Seg does not outperform all compared methods on every benchmark, the overall findings indicate that selective Mamba integration can be effective when combined with an appropriate placement strategy.

Future work may further investigate the mechanisms underlying placement-dependent behavior through effective receptive field and frequency-domain analyses, examine the influence of backbone initialization including training from scratch, explore additional lightweight state-space integration strategies, and evaluate the framework across broader medical image segmentation tasks and acquisition conditions.
